# Vitamin E Acetate Determination in Vaping Liquids and Non-targeted Analysis of Vaping Emissions of Diluents of Concern, Vitamin E Acetate and Medium-Chain Triglycerides Oil

**DOI:** 10.3389/fchem.2021.756745

**Published:** 2021-12-13

**Authors:** Ivana Kosarac, Cariton Kubwabo, Guru Prasad Katuri, Dora Petraccone, Trevor K. Mischki

**Affiliations:** ^1^ Office of Research and Surveillance, Tobacco Control Directorate, Controlled Substances and Cannabis Branch, Health Canada, Ottawa, ON, Canada; ^2^ Exposure and Biomonitoring Division, Environmental and Radiation Sciences Directorate, Healthy Environments and Consumer Safety Branch, Health Canada, Ottawa, ON, Canada

**Keywords:** vaping, vitamin E acetate, medium chain triglycerides, aerosol, nicotine, gc ms, emission, cannabis

## Abstract

During the summer of 2019, cases of lung injury associated with vaping emerged in North America, including among individuals who reported exclusive use of nicotine vaping liquids. Once vitamin E acetate was identified as a potential causative agent a quantitative method based on a simple sample dilution, separation by gas chromatography and analysis by triple quadrupole mass spectrometry (GC MSMS) was developed. Method detection limit (MDL) and limit of quantification (LOQ) were determined at 0.159 µg/mL and 0.505 µg/mL, respectively. The analysis was performed on a subset of 203 commercially sourced nicotine containing vaping liquids of various flavour profile and nicotine range (nicotine free-59 mg/mL) from an internal inventory. The target analyte, Vitamin E Acetate, was not detected in any samples analyzed, as expected, given the reported detection in literature and high association of the chemical with cannabis and not nicotine containing vaping products.

Emissions profiles of vitamin E acetate and Medium Chain Triglycerides (MCT) oil were also characterized *via* non-targeted analysis, using GC-MS/MS, to identify potential chemical analytes generated when liquids were heated. The emissions were generated under standardized conditions using a vaping machine and a vaporizer device designed for use with cannabis concentrates. Vitamin E acetate emissions mainly contained target analyte itself, while MCT oil emissions were more chemically complex, as expected due to the presence of various triglycerides in the un-vaped oil. Of note even following rigorous cleaning procedures of the device and contact parts with a number of chemical solvents and replacement of consumable parts between MCT oil and vitamin E acetate vaping sessions, cross-contamination was observed. This cross contamination and residue persistence, is indicative of a real-life scenario where users may share or use the same device with a number of different products or formulations and not be aware of potential exposures to diluents of concern.

## Introduction

E-cigarettes or vaping products are battery-powered, alternative nicotine delivery systems that vaporize a formulation of vaping liquid or e-liquid. The liquids most commonly contain carrier solvents, propylene glycol (PG) and/or vegetable glycerine (VG), nicotine and flavours. Once heated, the formulation generates additional chemical compounds in the emissions, resulting in a complex exposure pattern to users of these products. Nevertheless, in spite of potential health risks, vaping provides nicotine delivery with lower exposures to chemicals of concern for the individuals who smoke (Goniewicz et al., 2018). One of the main appealing characteristics of vaping products is the fact that they come in thousands of flavours and in various nicotine concentrations. Although some vaping products are available in a more closed, pod format, many products and flavour choices are available in open format. Users of open systems can refill or customize vaping liquids to be used, the most frequently, with a modifiable, custom power setting, vaping device. The vaping or heating principle is also used for delivery of other substances such as those containing cannabidiol (CBD) and tetrahydrocannabinol (THC) cannabis vaping products.

Vitamin E acetate or alpha tocopherol acetate is the stabilized form of vitamin E often used as food additive or nutrient supplement (National Library of Medicine 2021c). Medium chain triglycerides and vitamin E acetate are, reportedly, added to the vaping products in order to dilute the illicit THC-containing liquids and lower their costs. Moreover, diluents are also used in order to improve products’ appearance and flavour (Blount et al., 2020). Although ingestion of vitamin E acetate is thought to have nutritional and health benefits, the inhalation exposure studies in animals suggest a severe lung damage and impaired function (Bhat et al., 2020). Moreover, the recent studies on various diluents (coconut oil, medium-chain triglycerides, Vitamin E Acetate, etc) in *in-vitro* models have observed an increased cytotoxicity in human airway epithelial cells (Jiang et al., 2020) however, their effects in humans are still being investigated.

During the summer of 2019, cases of lung injury associated with vaping (vaping-associated lung illness (VALI (Canada)) or e-cigarette associated lung injury [EVALI (United States)] emerged in North America. In the course of investigation, illegal cannabis vaping products containing vitamin E acetate were found to be strongly associated and thought to be the most likely cause of the outbreak in the majority of cases in the United States (CDC 2020), as observed through laboratory analysis of patients’ biological and illicit THC product samples (Blount et al., 2020). MCT oil, to date, has not been reported as a cause of related adverse occurrence.

In 2019, when vitamin E acetate from the illicit products was identified as a potential causative agent of EVALI in the United States, new analytical method to quantify this target analyte in the Canadian vaping samples was developed. At the time, cannabis vaping products were illegal in Canada, so the analysis was carried out on the nicotine-containing products. Moreover, since the data on chemical transformations and constituents of emissions generated from heating of the pure vitamin E acetate were lacking, we characterized the same and identified chemicals relevant for the inhalation exposures. In addition to Vitamin E Acetate emission, MCT oil emission was characterized as well, and the main constituents are reported here.

## Materials and Methods

### Chemicals and Reagents

99.7% pure propylene glycol and 99.2% pure glycerol were purchased from Sigma-Aldrich (Oakville, Canada). HPLC grade methanol, acetonitrile and hexane were purchased from Fisher Scientific (Ottawa, Canada). Vitamin E acetate (D, L-α-Tocopherol acetate) (≥99%) analytical standard was purchased from Sigma-Aldrich (Oakville, Canada).

### Samples

Two hundred and three vaping liquids from over 70 manufacturers were collected between 2017 and 2019 from online and brick and mortar stores in Canada. These products were chosen at random from a larger pool of samples collected for a project on characterization of nicotine vaping liquids. The on-product labelled nicotine concentration varied between nicotine-free and 59 mg/mL (Supplementary Figure S1) and 20% of products were labelled to contain nicotine salts.

A majority of samples were packaged in glass or plastic 30 mL, refillable bottles and each one was classified into one of 18 flavour categories as per manufacturer’s declared flavour or based on product descriptions (Supplementary Figure S2). Flavour classification wheel was adapted for vaping products on Canadian market and based on previously reported approach (Krüsemann et al., 2019).

Medium Chain Triglyceride (MCT) oil from coconut oil (60% caprylic acid (C8)/40% capric acid (C10)-St. Francis, Herb Farm) was obtained from a local health food store in Ottawa, Canada. Vaping device used to generate emissions was Pax 3 (PAX, Labs) dual-use vaporizer device with concentrate insert consisting of aluminum oven purchased in vaping store in Ottawa, Canada.

### Chemical Analysis

Two analytical methods were developed one for targeted analysis and determination of concentration of vitamin E acetate in vaping liquids, and another one for characterization of emissions generated from MCT oil and vitamin E acetate.

### Targeted Analysis of Vaping Liquids

The sample preparation was a simple dilution in methanol. Following a thorough sample mixing, 40 mg of sample measured to the nearest 0.1 mg was diluted in a 20 mL volumetric flask and made up to volume with methanol. Upon dilution and vortex mixing, 1 µL of diluted sample was injected and analyzed using Thermo Ultra Trace GC (gas chromatograph) coupled to Quantum TSQ tandem mass spectrometer (MS/MS) (Thermo Elec. Missisausgua, Canada). The chromatographic separation was achieved on a Zebron ZB-1HT GC capillary column (15 × 0.25 mm x 0.1 µm) from Phenomenex (CA, United States). The injector temperature was set at 280°C and injection mode used was splitless. The GC oven temperature program was as follows: 120–200°C at 20°C/min, followed by 10°C/min to 290°C and holding for 1 min, and reaching 320°C at 20°C/min and finally holding for 3 min. Helium was the carrier gas at a flow rate of 1 mL/min in constant flow mode. The source and GC interface temperatures were set at 180 and 250°C, respectively. The mass spectrometer was operated in electron ionization at 70 eV in multiple-reaction monitoring (MRM) mode using argon as a collision gas. Xcalibur data system was used for data acquisition and processing. The following MRM transitions were optimized for vitamin E acetate quantifier ion: 430.3 → 165.05 with collision energy of 20 eV and qualifier ion: 472.1 → 430.75 with collision energy of 10 eV. The calibration was achieved using the standard addition method. Each individual calibration standard level was prepared using the aliquots of diluted sample and fortifying with target analyte, Vitamin E acetate. The calibration curve was linear in the concentration range 0.025-12.5 µg/mL.

### Method Performance and Validation

Method performance was assessed according to the EPA Regulation 40 CFR part 136 (Appendix B) method (USEPA 2011). Eight replicates of vaping sample with no detectable levels of vitamin E acetate were fortified with vitamin E acetate at 0.50 µg/mL, diluted with methanol and analyzed. The standard deviation associated with eight replicate analyses of fortified vaping liquid, was multiplied by the Student’s t value of 2.998 (appropriate for a 99% confidence level with 7 degrees of freedom). The method detection limit (MDL) for vitamin E acetate was calculated to be 0.159 µg/mL or 0.18 µg/g. The limit of quantitation (LOQ) was calculated according to the US EPA method, where the standard deviation associated with the eight replicate analyses of fortified vaping liquid was multiplied by a factor of 10. The LOQ was calculated to be 0.505 µg/mL.

Laboratory blanks consisting of methanol and vaping liquid containing *p*G/VG (50/50 v/v) prepared in laboratory were used to assess carry-over between injections and any possible contamination. Since the analytical methodology did not employ the use of internal standard nor extraction sample clean up, the measures of analyte recovery were irrelevant. However, using the standard addition calibration and quality control sample fortified at 0.50 µg/mL, processed as eight repeats on a single day and as a repeated QC sample in 4 analysed sample batches, accuracy and precision (%RSD) were determined as 6.9 and 18.1%, respectively.

#### Characterization of Vitamin E Acetate and Medium-Chain Triglycerides Oil, and Vaping Emissions

##### Emission Generation

Approximately 150 mg of vitamin E acetate or MCT oil were placed in the aluminum, concentrate, oven insert of a Pax 3 device. The mouthpiece was connected to a CETI 8 vaping machine (Cerulean, United States) and the Pax 3 was set to 215°C, (maximum device temperature setting). Generation of vaped emissions was achieved using the ISO 20768 puffing regimen of 55 ml square wave puff, over 3 s, once every 30 s ((ISO) 2018). Ten puffs of each vitamin E acetate or MCT oil were collected on solvent pre-cleaned collection pads. Device weight was recorded prior and after vaping. Collection pads were extracted twice with 5 mL of methanol. Extracts were combined and 1 µL of extract was injected and analyzed using GC MS.

##### Analysis of Diluents of Concern and Their Emissions

The Quantum TSQ MS/MS instrument coupled to a Trace GC Ultra gas chromatograph (Thermo Electron Corp.) was operated in a full-scan acquisition mode. The oven ramp programing was identical to the one used for targeted analysis of Vitamin E Acetate. The MS was operated in Electron Ionization, full-scan mode with scan range 35–600 m*/z* and emission current set at 100 µA. GC separation was performed using Zebron ZB-1HT GC capillary column (15 × 0.25 mm x 0.1 µm) from Phenomenex (CA, United States). The injector temperature was set at 280°C with splitless injection mode. GC carrier gas was helium operated in constant flow mode at 1 mL/min rate. The spectrum of detected compounds was matched against spectra from the National Institute of Standards and Technology (NIST 17) library reference peaks.

## Results and Discussion

### Vitamin E Acetate in Nicotine-Containing Vaping Liquids

**FIGURE 1:**
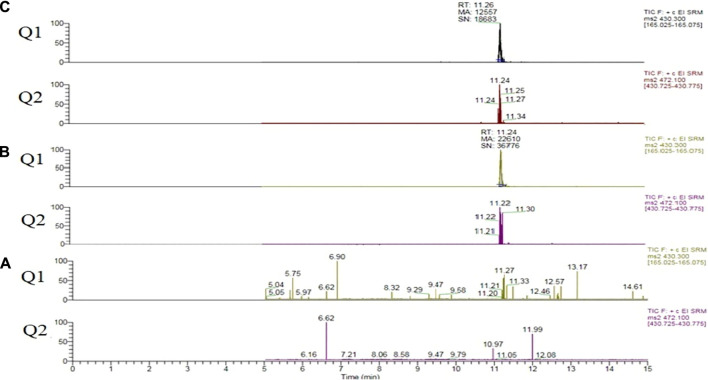
Vitamin E acetate extracted quantifier (Q1) and qualifier (Q2) ion transitions. **(A)** Sample 202. **(B)** Fortified sample 202 at 2.5 µg/mL of vitamin E acetate. **(C)** Pure vitamin E acetate diluted in methanol at 2.5 µg/mL (no matrix present).

During the development of the targeted method matrix effects were observed due to the presence of carrier solvents PG and VG, Figure 1.

In order to account for matrix effects the six level calibration was performed using the standard addition method, where diluted samples were fortified with known concentrations of Vitamin E Acetate. The analytical blank samples revealed no background contamination or in-between sample carry-over.

During the early reporting of EVALI, it was not clear which vaping product’s (nicotine or cannabis) constituents were related to the cases of the outbreak. Since the cannabis vaping products only became legal for sale in Canada in late 2019 (Government of Canada 2019) and would only have entered the marketplace towards mid-to end of December 2019 or early 2020, we screened the legal nicotine-containing products. The analytical method was applied to 203 nicotine-containing vaping liquids in order to quantify Vitamin E Acetate. The target analyte was not detected above limit of detection (0.159 µg/mL) in any products analyzed. To date and to our knowledge no nicotine-vaping liquids have been reported to contain Vitamin E Acetate. In fact nicotine-containing vaping products’ ingredient reports from cartridges and refill containers (41,809), were assessed for the presence of vitamin E acetate and THC by United Kingdom Centre for Tobacco and Alcohol Studies (Nyakutsikwa et al., 2020). Although no actual chemical analysis was conducted, the assessment of reported ingredients had found no Vitamin E Acetate and THC present. The chemical matrix of cannabis vaping liquids differs from that of nicotine containing vaping products due to distinct chemical properties of active ingredients added to the products. Cannabinoids are hydrophobic and typically diluted with oils or other, more hydrophobic diluents, while nicotine is diluted in more polar, alcohol-based diluents. Nicotine formulations in fact, often contain water (Crenshaw et al., 2016). While vitamin E acetate is a suspected cause of majority of EVALI cases in United States, more than half of cases reported in Canada appear to be associated with nicotine-containing vaping liquids (Government Of Canada 2020). Up to the August of 2020, 20 cases of VALI were reported in Canada, with 11 cases associated with nicotine containing vaping liquids only, however this is based on self-reporting and is not laboratory verified through testing of patients’ biological samples for presence of active substances (THC, nicotine, etc) or substances of concern (Vitamin E Acetate). The causes of these cases are an area of continued monitoring.

Reported analytical methods for the targeted analysis or quantification of vitamin E Acetate in vaping liquids and generated aerosols are limited, particularly for nicotine-containing products. In the VALI cases, mainly in the illicit cannabis vaping products, Vitamin E Acetate was quantified in relatively high concentrations since it was used as a diluent (Duffy et al., 2020). In analyzing such samples, analytical method’s detection sensitivity may not be of a concern; however, to date, toxicologically relevant concentrations of Vitamin E Acetate for inhalation exposures remain unknown; therefore, analytical methods that can detect the low levels of Vitamin E Acetate may be of interest as well. In a study by German federal institute for risk assessment (BfR), LC-MS/MS based method was developed to detect Vitamin E and Vitamin E Acetate in e-liquids with limit of detection for latter of 0.3 ng per Gram of e-liquid (The German Federal Institute for Risk Assessment (BfR) 2020). Although the method is more sensitive than method presented here (MDL = 18 ng per Gram of liquid), BfR method requires sample mass of 500 mg for analysis compared to 40 mg required in our methodology. Developing the methods that use small sample volume or weight is advantageous as often times, in the real cases of EVALI, forensic labs will only have a residue of vaping liquid, i.e. what remains in the device to be used for testing and not necessarily significant amounts of the product left. In other studies from the United States, LC-MS/MS methods were developed for analysis of Vitamin E Acetate in vaping products that contain tetrahydrocannabinol (THC) acquired from informal sources from Minnesota (Taylor et al., 2019) and in trapped aerosol emissions from e-cigarette, or vaping products associated with EVALI cases in United States (Puetz et al., 2021). Vitamin E acetate was also detected in THC cartridges and in bronchoalveolar lavage fluid from EVALI patients in Wisconsin United States (Pray et al., 2020). THC/VEA mixtures were vaped at elevated power levels in vaping device to study the VEA decomposition by GC/MS in Forensic Chemistry Center, United States (Lynch et al., 2021). Most of the reported studies lack detailed information on the method detection limits, validation data, sample preparation, and sample size.

### Medium Chain Triglyceride Oil

**FIGURE 2:**
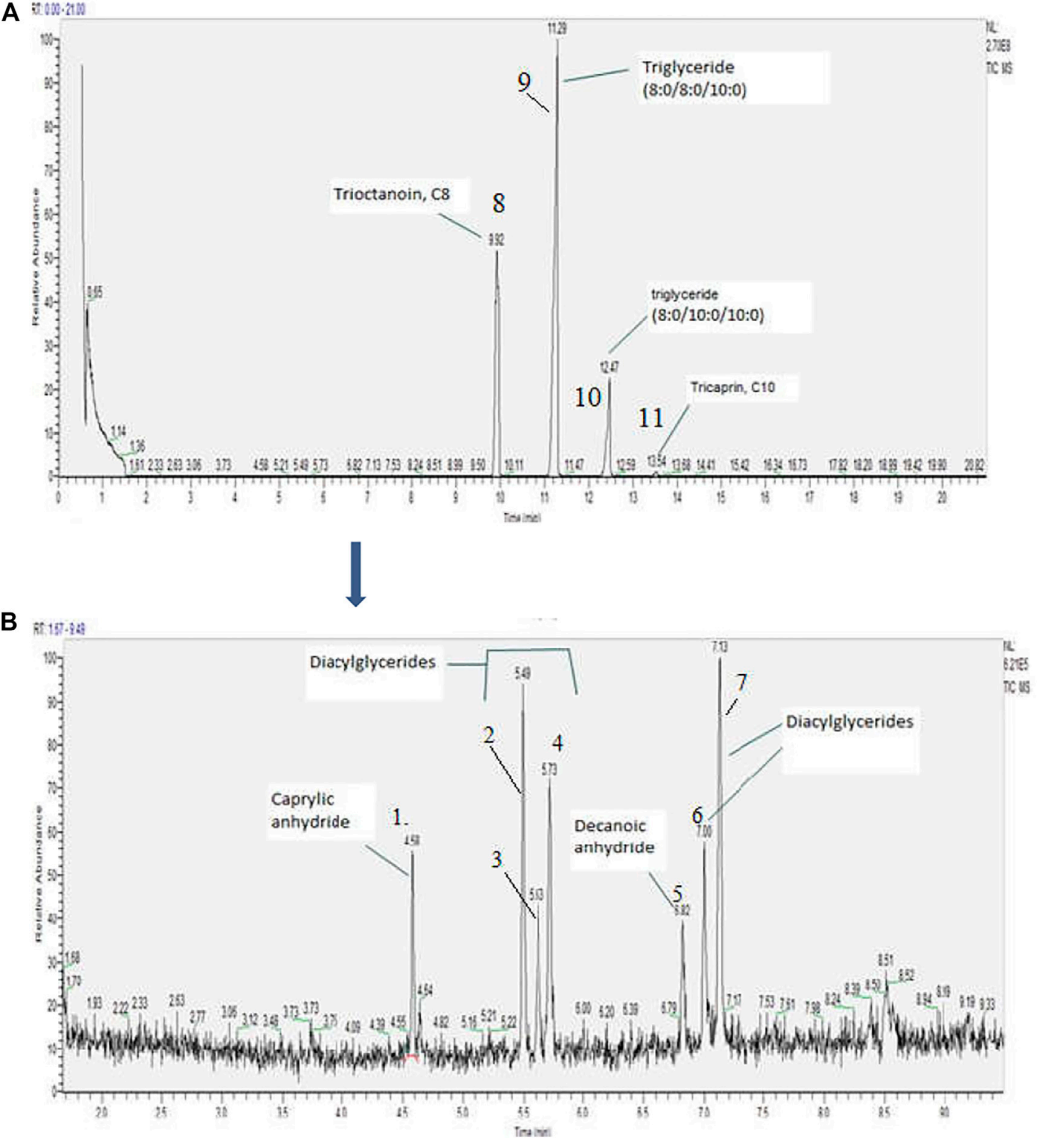
**(A)** MCT oil scan, concentration 2000 µg/mL; **(B)** Earlier eluting compounds area enlarged.

The saturated fatty acids of mixed triacylglycerols with 6–10 carbons chains are collectively referred to as medium chain triglyceride oil. MCT oil is included in a generally recognized as safe (GRAS) substances for human consumption (Traul et al., 2000). They are sourced from plant oil sources such as coconut and palm oils. This study used MCT oil from coconut which according to product label, was comprised of 60% caprylic acid (C8) and 40% capric acid (C10) triglycerides. Prior to heating and emission generation, chemical characterization of this oil was conducted to better understand the starting composition. Four major mixed triacylglyceride constituents were detected (Trioctanoin (8:0/8:0/8:0), glyceryl 2-caprate dicaprylate (8:0/8:0/10:0); glyceryl 2-caprylate dicaprate (10:0/10:0/8:0) and tricaprin (10:0/10:0/10:0), Figure 2A). In addition to major constituents, earlier eluting chemical compounds included two anhydrides caprylic and decanoic, as well as octanoic acid, 2-propenyl ester (imitation fatty, fruity, pineapple flavour agent) (The Good Scents Company 2021b) and four diacylglycerides, Figure 2B).

Table 1 contains detailed information on detected chemical compounds. Open source databases, Pubchem (National Library of Medicine 2021b), NIST Chemistry WebBook ((NIST) 2021) and The Good Scents Company Information System (The Good Scents Company 2021a) were used to extract more information about and typical use for detected chemicals.

**TABLE 1:** Chemical compounds detected in MCT oil (Refer to Figures 2, 3).

Peak	IUPAC Name	Common Name	CASRN	More information
1	Octanoyl octanoate	n-Caprylic Anhydride	623-66-5	Listed in cosmetic products patents
2	Prop-2-enyl octanoate	Octanoic acid, 2-propenyl ester	4230-97-1	Flavour and fragrance agent; Fatty, fruity, pineapple tropical-like flavour
3	[(2S)-3-hydroxy-2-octanoyloxypropyl] octanoate	Octanoic acid, 1-(hydroxymethyl)-1,2-ethanediyl ester, (S)-	60514-48-9	Common food additives used to blend together certain ingredients, such as oil and water, which would not otherwise blend well. Diacylglycerols are often found in bakery products, beverages, ice cream, chewing gum, shortening, whipped toppings, margarine, and confections
4	(2-hydroxy-3-octanoyloxypropyl) octanoate	1,3-Dioctanoin	1429-66-9	Metabolite of rapeseed
5	Decanoyl decanoate	Decanoic anhydride	2082-76-0	—
6	(1-hydroxy-3-octanoyloxypropan-2-yl) decanoate	Decanoic acid, 1-(hydroxymethyl)-2-[(1-oxooctyl)oxy]ethyl ester	177717-46-3	Patents list personal care product and emulsion formulations use
7	(2-hydroxy-3-octanoyloxypropyl) decanoate	Glyceryl 1-caprate-3-caprylate	93980-84-8	Patents list use in surfactants
8	2,3-di(octanoyloxy)propyl octanoate	Trioctanoin	538-23-8	Has a role as a plant metabolite. Used as diluent, as carrier for flavours and essential oils
9	1,3-di(octanoyloxy)propan-2-yl decanoate	Glyceryl 2-caprate dicaprylate	33368-87-5	Triglyceride 8:0/8:0/10:0, patents list use in printing inks and varnishes
10	(3-decanoyloxy-2-octanoyloxypropyl) decanoate	Glyceryl 2-caprylate dicaprate	33368-86-4	Triglyceride 8:0/10:0/10:0, patents list use in printing inks and varnishes
11	2,3-di(decanoyloxy)propyl decanoate	Tricaprin	621-71-6	Precursor of decanoic acid (DA), a 10-carbon fatty acid (Triglyceride 10:0/10:0/10:0) and major component of medium chain triglyceride oils. Used in dietary and cosmetic products as emollient and solvent

**FIGURE 3:**
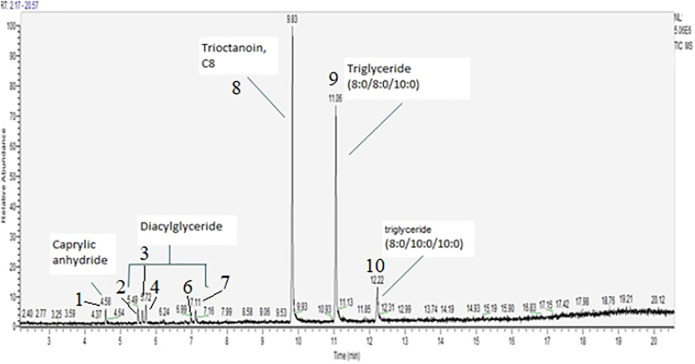
MCT oil emission scan, concentration 920 µg/ml.

Generated emissions of heated MCT oil in Pax 3 device were captured, extracted in methanol and analyzed (Figure 3). The analysis of ten puffs revealed presence of nine of eleven chemical compounds detected in the unvaped MCT oil.

Two chemicals that were not detected in the emissions were tricaprin (10:0/10:0/10:0 triglyceride), and decanoic anhydride. Comparing the chemical profile of unvaped MCT oil and compounds generated in the emissions it is evident that the absolute ratio of triglycerides changes, presumably due to the transformation during heating process or perhaps due to the varying degrees of transfer from oil into emissions given the differences in boiling points among the triglycerides. The exact concentrations of each generated chemical were not quantified as calibration and genuine analytical standards were not used. Instead, the overall concentration of MCT oil mixture was estimated using the mass loss before and after vaping and by weighing the collection pad.

Thinning agents such as vegetable glycerine, propylene glycol or poly ethylene glycol are used as diluents of cannabis vaping oils (Erickson 2019; Jiang et al., 2020). MCT oils have also been reported to be used in some cannabis products, however, to date and in comparison with Vitamin E Acetate, MCT oil has been less frequently detected in the samples associated with cases of EVALI in the United States (Blount et al., 2020).

### Vitamin E Acetate

The same procedure was followed with vitamin E acetate as with MCT oil. Vitamin E acetate was diluted in methanol and analyzed using the non-targeted or scanning protocol. This substance was of a high grade and purity as confirmed by the analysis where two isomers, dexter (D) and laevus (L) were detected as major compounds. Only one other compound was detected in the tested material and that is methyl stearate, flavouring and cosmetics agent as well as plant metabolite (National Library of Medicine 2021a), Figure 4 and Table 2.

**FIGURE 4:**
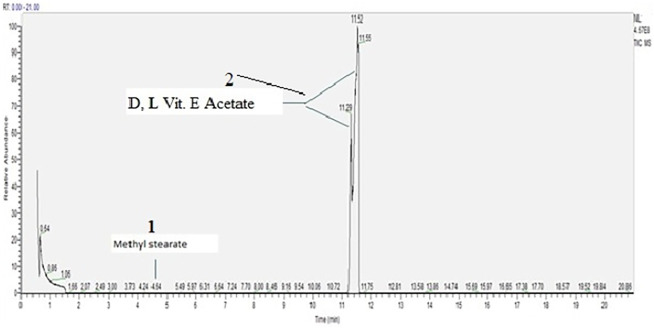
D, L Vitamin E acetate scan, concentration 2000 µg/ml.

**TABLE 2:** Chemical compounds detected in Vitamin E Acetate (Figure 4).

Peak	IUPAC Name	Common Name	CASRN	More information
1	Methyl octadecanoate	Methyl stearate	112-61-8	Found in cloves. Used as cosmetics and fragrance agent. Plant metabolite
2	[2,5,7,8-tetramethyl-2-(4,8,12-trimethyltridecyl)-3,4-dihydrochromen-6-yl] acetate	D,L Vitamin E acetate or (±)-α-Tocopherol acetate	7695-91-2	Synthetic D,L form. Antioxidant, nutritional supplement and preservative

**FIGURE 5:**
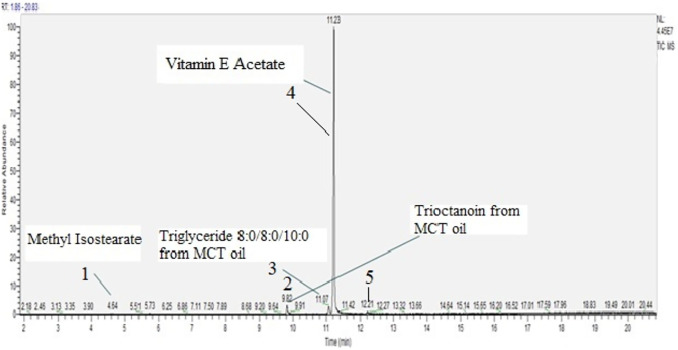
D, L vitamin E acetate emission scan, concentration 870 µg/mL, including two MCT oil cross-contaminants.

Aluminium oven from Pax 3 device was filled with Vitamin E Acetate, weight recorded and used to generate ten puffs on CETI 8 vaping machine. The emissions were collected on the chemically pre-cleaned collection pad which in turn was extracted with methanol and analyzed using the scanning protocol. It is important to note that the same Pax3 device was used to vape MCT oil first, and then D, L Vitamin E Acetate. Five chemical compounds were detected in D, L vitamin E acetate emission, Figure 5 and Table 3.

**TABLE 3:** Chemical compounds detected in vitamin E acetate emission (Figure 5).

Peak	IUPAC Name	Common Name	CASRN	More information
1	Methyl 16-methylheptadecanoate	Methyl isostearate	5129-61-3	Natural substance and extractive, used in cosmetic products
2	2,3-di(octanoyloxy)propyl octanoate	Trioctanoin	538-23-8	Has a role as a plant metabolite. Used as diluent, as carrier for flavours and essential oils
3	1,3-di(octanoyloxy)propan-2-yl decanoate	Glyceryl 2-caprate dicaprylate	33368-87-5	Triglyceride 8:0/8:0/10:0, patents list use in printing inks and varnishes
4	[2,5,7,8-tetramethyl-2-(4,8,12-trimethyltridecyl)-3,4-dihydrochromen-6-yl] acetate	D,L Vitamin E Acetate or (±)-α-Tocopherol acetate	7695-91-2	Synthetic D,L form. Antioxidant, nutritional supplement and preservative
5	(3-decanoyloxy-2-octanoyloxypropyl) decanoate	Glyceryl 2-caprylate dicaprate	33368-86-4	Triglyceride 8:0/10:0/10:0, patents list use in printing inks and varnishes

The major constituent of the emission was one racemic structure (D or L) of vitamin E acetate and a minor constituent was Methyl isostearate. Additional three chemicals detected in the emissions were most likely due to the cross-contamination with MCT oil emissions as they have been previously detected in the oil itself and its’ emission. Although in between the samples, the mouthpiece was replaced, an aluminium concentrate insert heating oven was thoroughly cleaned as per manufacturer’s instructions, and through the use of various laboratory chemical solvents (hexane, acetonitrile, methanol), the analysis of vitamin E acetate generated emissions, unfortunately, revealed the presence of MCT oil compounds as well. The cross-contamination did not result from the GC MS instrument carry over or instrument contamination as the analytical blanks injected between samples showed no detected MCT oil compounds. Although it was not confirmed through laboratory analysis, it is likely that the contamination originated from the heating element on the outside of the oven or from the other parts of the device that could have gotten into contact through emission path. Some interior device parts were not rinsed thoroughly with solvents as they could be damaged, and therefore, were a likely source of MCT oil triglycerides.

The cross contamination, although unfortunate, can be indicative of a real-life occurrence where users may use the same device to vaporize or vape a number of different products or formulations, and unintentionally get exposed to chemical transformation products that may not necessarily match vaping liquid they are consuming in spite of a thorough device cleaning. Of a particular concern may also be the cases where a device is shared or borrowed among users - a social behaviour previously documented among youth who vape (Pepper et al., 2019). In such cases, cross-contamination or persistence of diluent of concern in the vaping device may lead to lack of awareness and unintentional exposure of additional users.

### Limitations

The vitamin E acetate targeted method presented here was not applied to any cannabis-containing vaping products, therefore, no conclusions can be made on presence of this diluent in cannabis products in Canada. Vitamin E acetate (D, L) emissions in our study were generated using 96% pure compound. The chemical grade of vitamin E acetate used as diluent for illicit cannabis vaping products may contain more impurities and be of a lower grade. In the absence of real cannabis sample or type of diluent used, it is difficult to conclude whether the impurities from the technical grade product would be present in the emissions.

While this study provides an improved understanding of chemical profiles of emissions of vaping diluents, caution should be taken when interpreting these results in real-world conditions. In order to study formation of chemical transformations, the liquid diluents were studied individually and not in vaping liquid mixtures which most frequently will include other diluents (PG or VG), flavours, processing agents and active ingredients (nicotine, THC, etc).

Recent studies on pyrolysis of vitamin E acetate have hypothesized and detected a presence of a simple ketene-ethenone, an acute lung irritant and highly reactive compound (Wu and O’Shea 2020). We did not detect this chemical compound in the emissions as it is likely that our pad capturing method was not sufficient to collect this highly volatile compound. Another reason could also be that the device used in our study was heating only to 215°C that may not have been sufficient temperature to generate this compound (Attfield et al., 2020). Further studies are required to capture and characterize the highly volatile chemical compounds generated in the vaped emissions.

## Conclusion

In our study, Vitamin E Acetate was not detected above detection limit (0.159 µg/mL) in any nicotine containing vaping liquids analyzed. Two diluents of concern-Vitamin E Acetate and medium chain triglycerides, were analyzed as pure substances in order to better characterize their emissions. Both diluents when heated in vaping device in fact, generate high concentrations of diluents as well as other chemical compounds typically present in unvaped precursor material. Of a note is that there was an observed cross-contamination of vaping device with diluents used which may have implications for device sharing. Although Vitamin E Acetate has been identified as a potential causative agent in the occurrence of EVALI, MCT oil, to date has not been directly implicated in EVALI. Moreover, to date the cause of cases related to the exclusive use of nicotine-containing vaping products has not been determined. Future studies should characterize vaped emissions from the products of interest in order to better elucidate chemicals of concern.

## Data Availability

The original contributions presented in the study are included in the article/Supplementary Material, further inquiries can be directed to the corresponding author.
