# Supplementary figures and images for: Vitamin E Acetate Determination in Vaping Liquids and Non-targeted Analysis of Vaping Emissions of Diluents of Concern, Vitamin E Acetate and Medium-Chain Triglycerides Oil
Source: Front Chem. 2021 Dec 13;9:756745. doi: 10.3389/fchem.2021.756745 (PMC8710514; doi:10.3389/fchem.2021.756745)

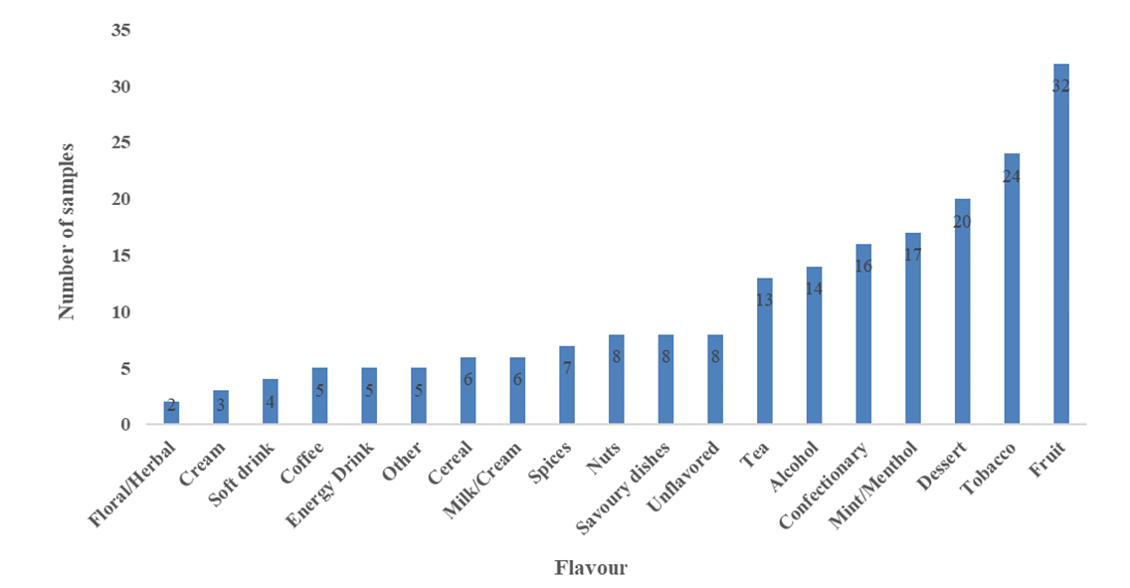

Supplement: Supplementary file 1 [file Image2.TIF]

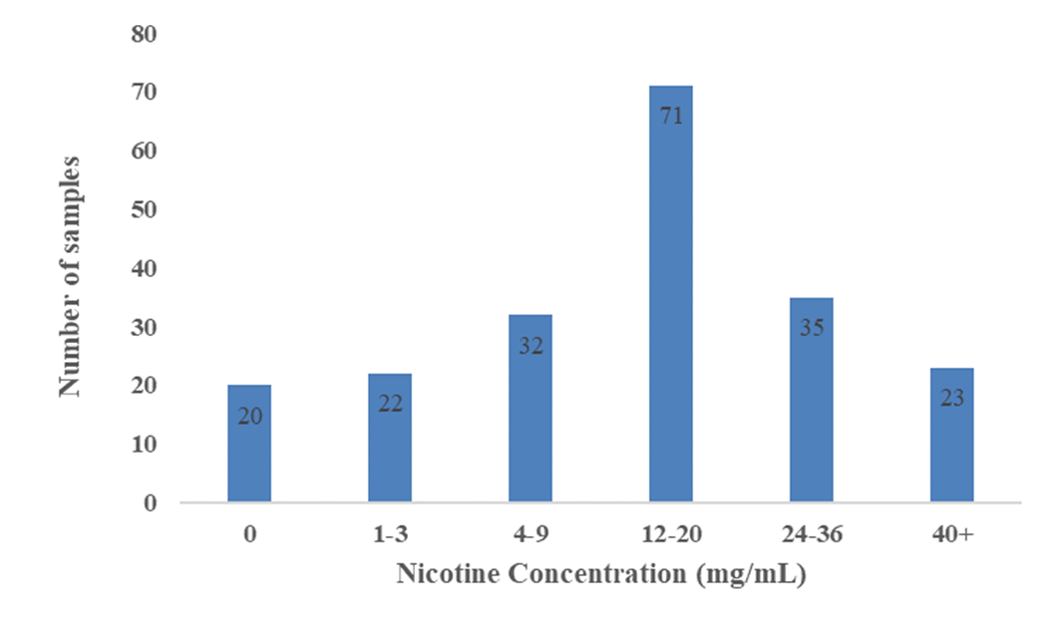

Supplement: Supplementary file 2 [file Image1.TIF]
